# The genome sequence of the oak bush-cricket, *Meconema thalassinum *(De Geer, 1773)

**DOI:** 10.12688/wellcomeopenres.21500.1

**Published:** 2024-06-28

**Authors:** Liam M. Crowley, Scott Hotaling, University of Oxford and Wytham Woods Genome Acquisition Lab, Darwin Tree of Life Barcoding collective, Wellcome Sanger Institute Tree of Life Management, Samples and Laboratory team, Wellcome Sanger Institute Scientific Operations: Sequencing Operations, Wellcome Sanger Institute Tree of Life Core Informatics team, Tree of Life Core Informatics collective, Darwin Tree of Life Consortium

**Affiliations:** 1 Department of Biology, University of Oxford, Oxford, England, UK; 2 School of Biological Sciences, Washington State University, Pullman, Washington, USA

**Keywords:** Meconema thalassinum, oak bush-cricket, genome sequence, chromosomal, Orthoptera

## Abstract

We present a genome assembly from one male *Meconema thalassinum* (the oak bush-cricket; Arthropoda; Insecta; Orthoptera; Tettigoniidae). The genome sequence is 9,039.1 megabases in span. Most of the assembly is scaffolded into 15 chromosomal pseudomolecules, including the X sex chromosome. The mitochondrial genome has also been assembled and is 15.63 kilobases in length.

## Species taxonomy

Eukaryota; Opisthokonta; Metazoa; Eumetazoa; Bilateria; Protostomia; Ecdysozoa; Panarthropoda; Arthropoda; Mandibulata; Pancrustacea; Hexapoda; Insecta; Dicondylia; Pterygota; Neoptera; Polyneoptera; Orthoptera; Ensifera; Tettigoniidea; Tettigonioidea; Tettigoniidae; Meconematinae; *Meconema*; *Meconema thalassinum* (De Geer, 1773) (NCBI:txid494438).

## Background

The Oak Bush-cricket ( *Meconema thalassinum*) is a small, pale green bush cricket in the family Tettigoniidae. It has long antennae, a pale cream/yellow dorsal stripe and the adults of both sexes are fully winged. It is native to Europe and has established itself in North America over the last approximately 75 years ( Cannings *et al.*, 2007). In the UK it is widespread and common across southern and central England and south Wales. *M. thalassinum* is the only native arboreal Orthopteran species in the UK, although the recently established Southern Oak Bush Cricket, *Meconema meridionale*, is also arboreal. Whilst similar in appearance, *M. meridionale* can be distinguished by the reddish marking on the prothorax and brachypterous adults. The Oak Bush-cricket is primarily associated with mature trees of a range of species, but in particular oaks. It is nocturnal and unlike other bush-crickets, Meconema species are carnivorous, feeding a range of invertebrates including Lepidopteran larvae ( Vahed, 1996). Males also has a unique auditory method for attracting females by drumming their hind limbs on leaves ( Sismondo, 1980). This is unusual for the Tettigoniidae, as most species typically have a stridulatory apparatus. Still, *M. thalassinum* produce some of the more complex sounds for the group ( Sismondo, 1980). Adults are present from late July to the autumn and eggs are laid in crevices in bark.

As a genomic resource, the *M. thalassinum* assembly is important in many ways. It is one of few high-quality assemblies for the order Orthoptera and the first for the family Tettigoniidae ( Hotaling *et al.*, 2021b). It also bolsters arthropod genomic resources broadly which are severely underrepresented in animal genome science ( Hotaling *et al.*, 2021a). Finally, it provides a key reference for studies seeking the genomic basis of acoustic behaviours in insects and/or to understand why some lineages have higher capacities for invading new regions than others.

The genome of the Oak Bush-cricket, *Meconema thalassinum*, was sequenced as part of the Darwin Tree of Life Project, a collaborative effort to sequence all named eukaryotic species in the Atlantic Archipelago of Britain and Ireland.

## Genome sequence report

**Figure 1.**
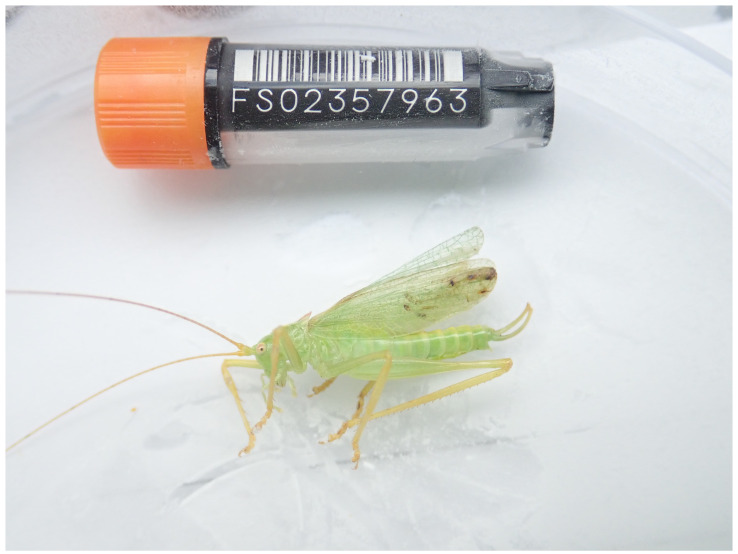
Photograph of the *Meconema thalassinum* (iqMecThal1) specimen used for genome sequencing.

The genome was sequenced from a male *Meconema thalassinum* ( Figure 1) collected from Wytham Woods, Oxfordshire, UK (51.77, –1.33). A total of 29-fold coverage in Pacific Biosciences single-molecule HiFi long reads and 33-fold coverage in 10X Genomics read clouds was generated. Primary assembly contigs were scaffolded with chromosome conformation Hi-C data. Manual assembly curation corrected 260 missing joins or mis-joins and removed 3 haplotypic duplications, reducing the scaffold number by 21.14%, and also decreasing the scaffold N50 by 19.95%.

**Figure 2.**
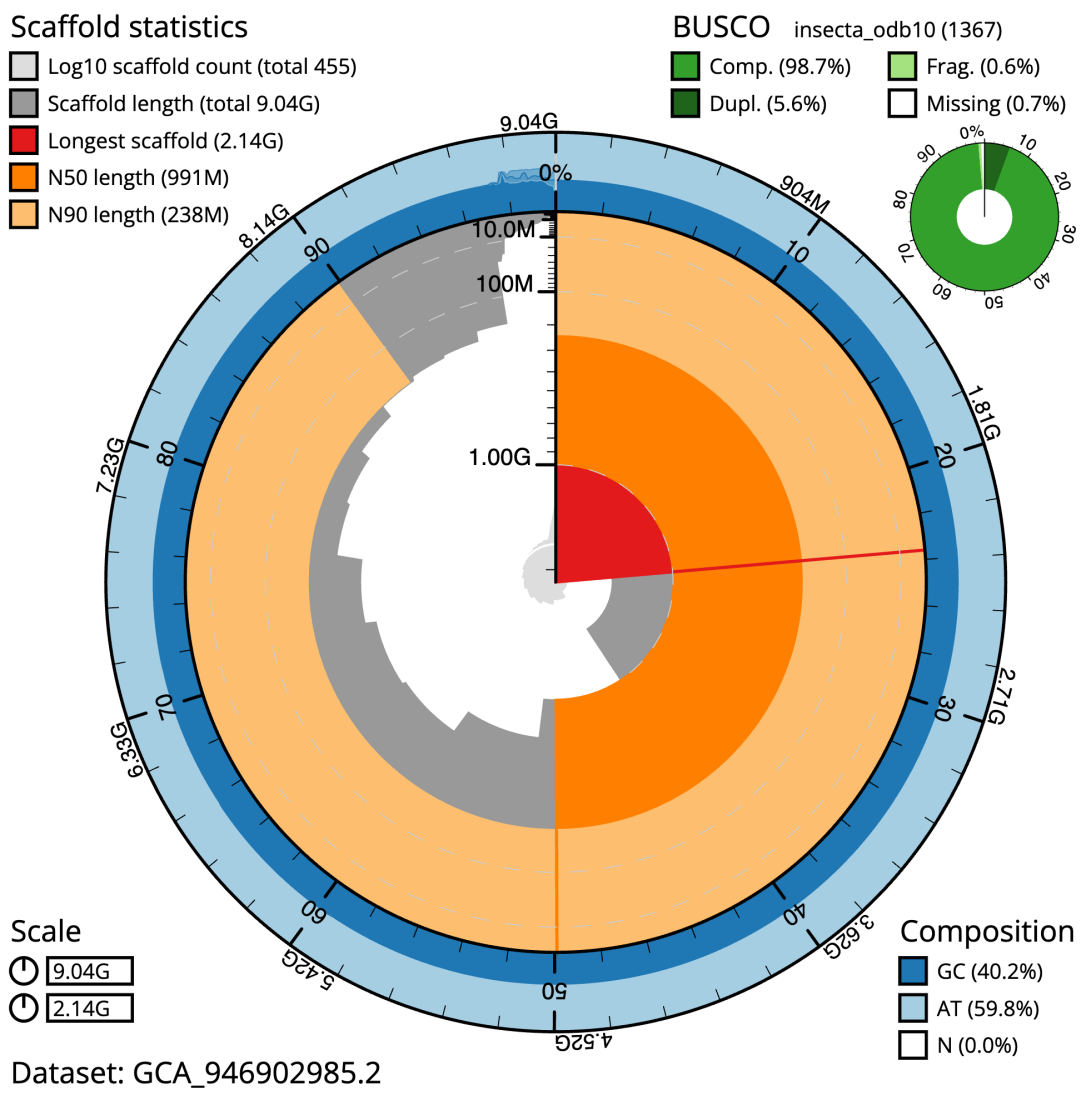
Genome assembly of *Meconema thalassinum*, iqMecThal1.2: metrics. The BlobToolKit snail plot shows N50 metrics and BUSCO gene completeness. The main plot is divided into 1,000 size-ordered bins around the circumference with each bin representing 0.1% of the 9,039,075,905 bp assembly. The distribution of scaffold lengths is shown in dark grey with the plot radius scaled to the longest scaffold present in the assembly (2,140,038,457 bp, shown in red). Orange and pale-orange arcs show the N50 and N90 scaffold lengths (991,394,496 and 237,816,702 bp), respectively. The pale grey spiral shows the cumulative scaffold count on a log scale with white scale lines showing successive orders of magnitude. The blue and pale-blue area around the outside of the plot shows the distribution of GC, AT and N percentages in the same bins as the inner plot. A summary of complete, fragmented, duplicated and missing BUSCO genes in the insecta_odb10 set is shown in the top right. An interactive version of this figure is available at https://blobtoolkit.genomehubs.org/view/Meconema%20thalassinum/dataset/GCA_946902985.2/snail.

**Figure 3.**
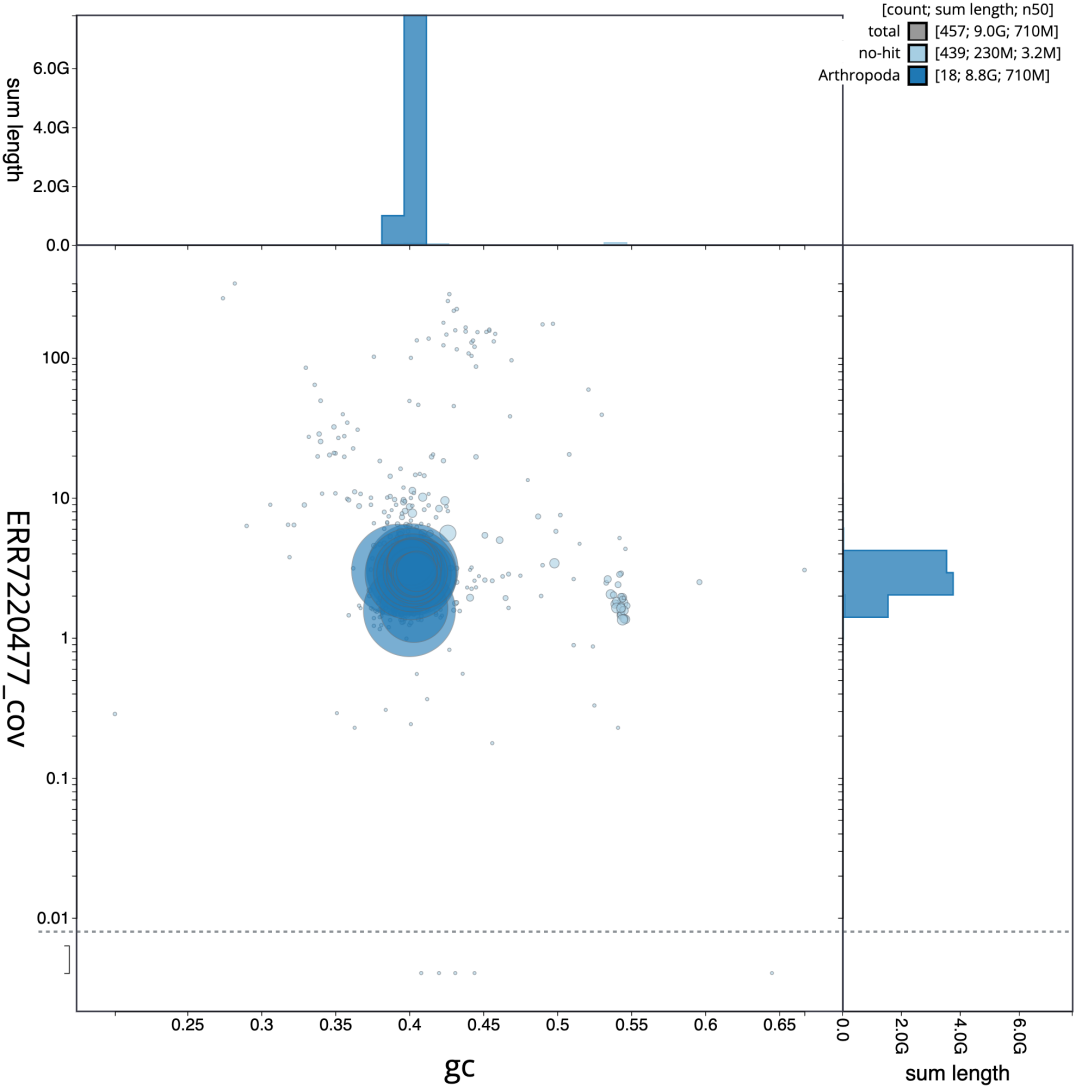
Genome assembly of *Meconema thalassinum*, iqMecThal1.2: BlobToolKit GC-coverage plot. Sequences are coloured by phylum. Circles are sized in proportion to sequence length. Histograms show the distribution of sequence length sum along each axis. An interactive version of this figure is available at https://blobtoolkit.genomehubs.org/view/CAMPQE01/dataset/CAMPQE01/blob.

**Figure 4.**
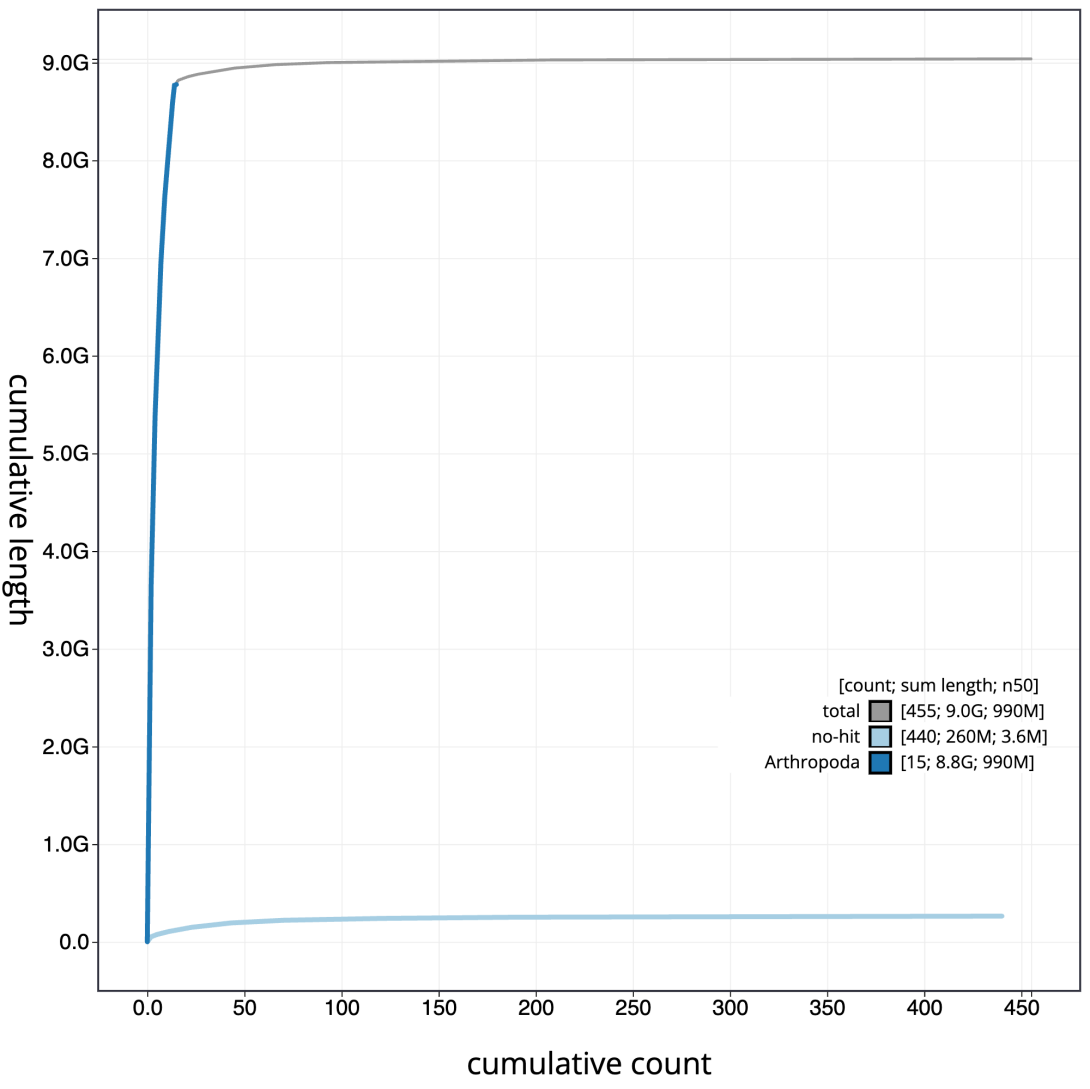
Genome assembly of *Meconema thalassinum*, iqMecThal1.2: BlobToolKit cumulative sequence plot. The grey line shows cumulative length for all sequences. Coloured lines show cumulative lengths of sequences assigned to each phylum using the buscogenes taxrule. An interactive version of this figure is available at https://blobtoolkit.genomehubs.org/view/Meconema%20thalassinum/dataset/GCA_946902985.2/cumulative.

**Figure 5.**
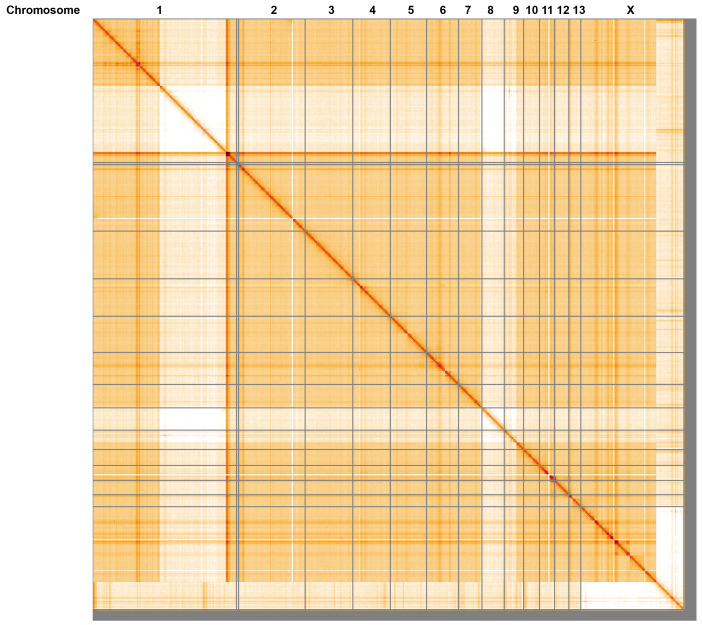
Genome assembly of *Meconema thalassinum*, iqMecThal1.2: Hi-C contact map of the iqMecThal1.2 assembly, visualised using HiGlass. An interactive version of this figure may be viewed at https://genome-note-higlass.tol.sanger.ac.uk/l/?d=ZcNIcdI5Ra2NliHtKeZaAQ.

The final assembly has a total length of 9,039.1 Mb in 454 sequence scaffolds with a scaffold N50 of 991.4 Mb ( Table 1). The snail plot in Figure 2 provides a summary of the assembly statistics, while the distribution of assembly scaffolds on GC proportion and coverage is shown in Figure 3. The cumulative assembly plot in Figure 4 shows curves for subsets of scaffolds assigned to different phyla. Most (98.69%) of the assembly sequence was assigned to 15 chromosomal-level scaffolds, representing 13 autosomes and the X sex chromosome. This specimen is a XO male. Chromosome-scale scaffolds confirmed by the Hi-C data shown on the HiGlass contact map ( Figure 5; Table 2). The order and orientation of scaffolds are uncertain on chromosome 1 in the region 1.647–1.695 Gb. While not fully phased, the assembly deposited is of one haplotype. Contigs corresponding to the second haplotype have also been deposited. The mitochondrial genome was also assembled and can be found as a contig within the multifasta file of the genome submission.

**Table 1.** Genome data for *Meconema thalassinum*, iqMecThal1.2.

Project accession data		
Assembly identifier	iqMecThal1.2	
Species	*Meconema thalassinum*	
Specimen	iqMecThal1	
NCBI taxonomy ID	494438	
BioProject	PRJEB48399	
BioSample ID	SAMEA7520379	
Isolate information	iqMecThal1 iqMecThal1	
Assembly metrics *		*Benchmark*
Consensus quality (QV)	62.8	*≥ 50*
*k*-mer completeness	100.0%	*≥ 95%*
BUSCO **	C:98.8%[S:93.2%,D:5.6%],F:0.6%, M:0.6%,n:1,367	*C ≥ 95%*
Percentage of assembly mapped to chromosomes	98.69%	*≥ 95%*
Sex chromosomes	XO	*localised homologous pairs*
Organelles	Mitochondrial genome: 15.63 kb	*complete single alleles*
Raw data accessions		
PacificBiosciences SEQUEL II	ERR7254641, ERR7254642, ERR7254646, ERR7254650, ERR7254643, ERR7254647, ERR7254648, ERR7254649, ERR7254651, ERR7254640, ERR7254644, ERR7254645, ERR7254652	
Hi-C Illumina	ERR7220492	
Genome assembly		
Assembly accession	GCA_946902985.2	
*Accession of alternate* *haplotype*	GCA_943193665.1	
Span (Mb)	9,039.1	
Number of contigs	2252	
Contig N50 length (Mb)	9.9	
Number of scaffolds	454	
Scaffold N50 length (Mb)	991.4	
Longest scaffold (Mb)	2,173.7	

* Assembly metric benchmarks are adapted from column VGP-2020 of “Table 1: Proposed standards and metrics for defining genome assembly quality” from Rhie *et al.* (2021). ** BUSCO scores based on the insecta_odb10 BUSCO set using version 5.3.2. C = complete [S = single copy, D = duplicated], F = fragmented, M = missing, n = number of orthologues in comparison. A full set of BUSCO scores is available at https://blobtoolkit.genomehubs.org/view/CAMPQE01/dataset/CAMPQE01/busco.

**Table 2.** Chromosomal pseudomolecules in the genome assembly of *Meconema thalassinum*, iqMecThal1.

INSDC accession	Chromosome	Length (Mb)	GC%
OX336388.2	1_1	2,140.04	39.5
OY714911.1	1_2	30.54	41.0
OX336390.1	2	991.39	40.0
OX336391.1	3	709.21	40.0
OX336392.1	4	559.01	41.0
OX336393.1	5	538.61	40.0
OX336394.1	6	476.62	40.5
OX336395.1	7	348.47	40.5
OX336396.1	8	332.26	40.5
OX336397.1	9	287.43	40.0
OX336398.1	10	237.82	40.0
OX336399.1	11	222.57	40.5
OX336400.1	12	213.54	40.5
OX336401.1	13	178.04	40.5
OX336389.2	X	1,533.31	40.0
OY714912.1	MT	0.02	28.0

The estimated Quality Value (QV) of the final assembly is 62.8 with *k*-mer completeness of 100.0%, and the assembly has a BUSCO v5.3.2 completeness of 98.8% (single = 93.2%, duplicated = 5.6%), using the insecta_odb10 reference set ( *n* = 1,367).

Metadata for specimens, barcode results, spectra estimates, sequencing runs, contaminants and pre-curation assembly statistics are given at https://links.tol.sanger.ac.uk/species/494438.

## Methods

### Sample acquisition and nucleic acid extraction

A male *Meconema thalassinum* (specimen ID Ox000173, ToLID iqMecThal1) was collected from Wytham Woods, Oxfordshire (biological vice-county Berkshire), UK (latitude 51.77, longitude –1.33) on 2019-08-13 by potting. The specimen was collected and identified by Liam Crowley (University of Oxford) and preserved on dry ice.

The workflow for high molecular weight (HMW) DNA extraction at the Wellcome Sanger Institute (WSI) Tree of Life Core Laboratory includes a sequence of core procedures: sample preparation; sample homogenisation, DNA extraction, fragmentation, and clean-up. In sample preparation, the iqMecThal1 sample was weighed and dissected on dry ice ( Jay *et al.*, 2023). Tissue from the abdomen and thorax was homogenised using a PowerMasher II tissue disruptor ( Denton *et al.*, 2023a).

HMW DNA was extracted using the Manual MagAttract v1 protocol ( Strickland *et al.*, 2023b). DNA was sheared into an average fragment size of 12–20 kb in a Megaruptor 3 system ( Todorovic *et al.*, 2023). Sheared DNA was purified by solid-phase reversible immobilisation ( Strickland *et al.*, 2023a): in brief, the method employs a 1.8X ratio of AMPure PB beads to sample to eliminate shorter fragments and concentrate the DNA. The concentration of the sheared and purified DNA was assessed using a Nanodrop spectrophotometer and Qubit Fluorometer and Qubit dsDNA High Sensitivity Assay kit. Fragment size distribution was evaluated by running the sample on the FemtoPulse system.

Protocols developed by the WSI Tree of Life laboratory are publicly available on protocols.io ( Denton *et al.*, 2023b).

### Sequencing

Pacific Biosciences HiFi circular consensus DNA sequencing libraries were constructed according to the manufacturers’ instructions. DNA sequencing was performed by the Scientific Operations core at the WSI on a Pacific Biosciences SEQUEL II instrument. Hi-C data were also generated from head tissue of iqMecThal1 using the Arima2 kit, following the manufacturer’s protocol, and sequenced on the Illumina NovaSeq 6000 instrument.

### Genome assembly and curation

Assembly was carried out with Hifiasm ( Cheng *et al.*, 2021) and haplotypic duplication was identified and removed with purge_dups ( Guan *et al.*, 2020). The assembly was then scaffolded with Hi-C data ( Rao *et al.*, 2014) using YaHS ( Zhou *et al.*, 2023). The assembly was checked for contamination and corrected as described previously ( Howe *et al.*, 2021). Manual curation was performed using HiGlass ( Kerpedjiev *et al.*, 2018) and PretextView ( Harry, 2022). The mitochondrial genome was assembled using MitoHiFi ( Uliano-Silva *et al.*, 2023), which runs MitoFinder ( Allio *et al.*, 2020) or MITOS ( Bernt *et al.*, 2013) and uses these annotations to select the final mitochondrial contig and to ensure the general quality of the sequence.

### Assembly evaluation

The final assembly was post-processed and evaluated with the three Nextflow ( Di Tommaso *et al.*, 2017) DSL2 pipelines “sanger-tol/readmapping” ( Surana *et al.*, 2023a), “sanger-tol/genomenote” ( Surana *et al.*, 2023b) and "sanger-tol/blobtoolkit" ( Muffato *et al.*, 2024).

A Hi-C map for the final assembly was produced using bwa-mem2 ( Vasimuddin *et al.*, 2019) in the Cooler file format ( Abdennur & Mirny, 2020). To assess the assembly metrics, the *k*-mer completeness and QV consensus quality values were calculated in Merqury ( Rhie *et al.*, 2020).

The pipeline “sanger-tol/blobtoolkit” is a Nextflow port of the existing Snakemake pipeline ( Challis *et al.*, 2020). It aligns the PacBio reads with samtools ( Danecek *et al.*, 2021) and minimap2 ( Li, 2018) and generates coverage tracks for regions of fixed size. In parallel, it queries the GoaT database ( Sotero-Caio *et al.*, 2021) to identify all matching BUSCO lineages to run BUSCO ( Manni *et al.*, 2021; Simão *et al.*, 2015). For the three domain-level BUSCO lineage, the pipeline aligns the BUSCO genes to the Uniprot Reference Proteomes database ( Bateman *et al.*, 2023) with DIAMOND ( Buchfink *et al.*, 2021) blastp. The genome is also split into chunks according to the density of the BUSCO genes from the closest taxonomically lineage, and each chunk is aligned to the Uniprot Reference Proteomes database with DIAMOND blastx. Genome sequences that have no hit are then chunked with seqtk and aligned to the NT database with blastn ( Altschul *et al.*, 1990). All those outputs are combined with the blobtools suite into a blobdir for visualisation.

Table 3 contains a list of relevant software tool versions and sources.

**Table 3.** Software: versions and sources.

Software tool	Version	Source
BEDTools	2.30.0	https://github.com/arq5x/bedtools2
Blast	2.14.0	ftp://ftp.ncbi.nlm.nih.gov/blast/executables/blast+/
BUSCO	5.4.3 and 5.5.0	https://gitlab.com/ezlab/busco
bwa-mem2	2.2.1	https://github.com/bwa-mem2/bwa-mem2
Cooler	0.8.11	https://github.com/open2c/cooler
Datasets	15.12.0	https://github.com/ncbi/datasets
DIAMOND	2.1.8	https://github.com/bbuchfink/diamond
fasta_windows	0.2.4	https://github.com/tolkit/fasta_windows
FastK	427104ea91c78c3b8b8b49f1a7d6bbeaa869ba1c	https://github.com/thegenemyers/FASTK
GoaT CLI	0.2.5	https://github.com/genomehubs/goat-cli
Hifiasm	0.15.3	https://github.com/chhylp123/hifiasm
HiGlass	44086069ee7d4d3f6f3f0012569789ec138f42b84 aa44357826c0b6753eb28de	https://github.com/higlass/higlass
MerquryFK	d00d98157618f4e8d1a9190026b19b471055b2 2e	https://github.com/thegenemyers/MERQURY.FK
MitoHiFi	2	https://github.com/marcelauliano/MitoHiFi
MultiQC	1.14, 1.17, and 1.18	https://github.com/MultiQC/MultiQC
Nextflow	23.04.0-5857	https://github.com/nextflow-io/nextflow
PretextView	0.2	https://github.com/wtsi-hpag/PretextView
purge_dups	1.2.3	https://github.com/dfguan/purge_dups
samtools	1.16.1, 1.17, and 1.18	https://github.com/samtools/samtools
sanger-tol/blobtoolkit	0.3.0	https://github.com/sanger-tol/blobtoolkit
sanger-tol/genomenote	1.0	https://github.com/sanger-tol/genomenote
sanger-tol/readmapping	1.1.0	https://github.com/sanger-tol/readmapping/tree/1.1.0
seqtk	1.3	https://github.com/lh3/seqtk
Singularity	3.9.0	https://github.com/sylabs/singularity
YaHS	1	https://github.com/c-zhou/yahs

### Wellcome Sanger Institute – Legal and Governance

The materials that have contributed to this genome note have been supplied by a Darwin Tree of Life Partner. The submission of materials by a Darwin Tree of Life Partner is subject to the **‘Darwin Tree of Life Project Sampling Code of Practice’**, which can be found in full on the Darwin Tree of Life website here. By agreeing with and signing up to the Sampling Code of Practice, the Darwin Tree of Life Partner agrees they will meet the legal and ethical requirements and standards set out within this document in respect of all samples acquired for, and supplied to, the Darwin Tree of Life Project.

Further, the Wellcome Sanger Institute employs a process whereby due diligence is carried out proportionate to the nature of the materials themselves, and the circumstances under which they have been/are to be collected and provided for use. The purpose of this is to address and mitigate any potential legal and/or ethical implications of receipt and use of the materials as part of the research project, and to ensure that in doing so we align with best practice wherever possible. The overarching areas of consideration are:

•   Ethical review of provenance and sourcing of the material

•   Legality of collection, transfer and use (national and international)

Each transfer of samples is further undertaken according to a Research Collaboration Agreement or Material Transfer Agreement entered into by the Darwin Tree of Life Partner, Genome Research Limited (operating as the Wellcome Sanger Institute), and in some circumstances other Darwin Tree of Life collaborators.

## Data Availability

European Nucleotide Archive: *Meconema thalassinum* (oak bush-cricket). Accession number PRJEB48399; https://identifiers.org/ena.embl/PRJEB48399 ( Wellcome Sanger Institute, 2023). The genome sequence is released openly for reuse. The *Meconema thalassinum* genome sequencing initiative is part of the Darwin Tree of Life (DToL) project. All raw sequence data and the assembly have been deposited in INSDC databases. The genome will be annotated using available RNA-Seq data and presented through the Ensembl pipeline at the European Bioinformatics Institute. Raw data and assembly accession identifiers are reported in Table 1. Members of the University of Oxford and Wytham Woods Genome Acquisition Lab are listed here: https://doi.org/10.5281/zenodo.7125292. Members of the Darwin Tree of Life Barcoding collective are listed here: https://doi.org/10.5281/zenodo.4893703. Members of the Wellcome Sanger Institute Tree of Life Management, Samples and Laboratory team are listed here: https://doi.org/10.5281/zenodo.10066175. Members of Wellcome Sanger Institute Scientific Operations: Sequencing Operations are listed here: https://doi.org/10.5281/zenodo.10043364. Members of the Wellcome Sanger Institute Tree of Life Core Informatics team are listed here: https://doi.org/10.5281/zenodo.10066637. Members of the Tree of Life Core Informatics collective are listed here: https://doi.org/10.5281/zenodo.5013541. Members of the Darwin Tree of Life Consortium are listed here: https://doi.org/10.5281/zenodo.4783558.
