# Supplementary material for: The genome sequence of the oak bush-cricket, Meconema thalassinum (De Geer, 1773)
Source: Wellcome Open Res. 2024 Jun 28;9:346. [Version 1] doi: 10.12688/wellcomeopenres.21500.1 (PMC11467643; doi:10.12688/wellcomeopenres.21500.1)
Supplement: Peer review history

# Reviewer report: Reviewer response for version 1

**DOI:** 10.21956/wellcomeopenres.23770.r98398

**Published:** 2024-10-10

**Authors:** James Pflug

**Affiliations:** 1 Oregon State University, Corvallis, Oregon, USA

The paper provides a sound justification for the choice of *M. thalassinum* for genome sequencing, and effectively summarises the process used to acquire and analyse the data. All figures and tables were clear and pertinent to the text, and I was able to easily locate all relevant data and externally hosted supplementary information. The methods were understandable and concise, and the most important results were clearly stated.

 I have no major criticisms or suggestions, and I believe the paper is more than adequate for indexing in its current form. I only have two minor suggestions for additional information/clarification, and neither are not essential to the quality of the overall paper

 A sentence or two mentioning any known or potential ecological impacts of this cricket’s introduction to North America would be useful additional information and provide further justification for the choice to sequence the genome of this species.    

 I would also find it useful to include a brief mention/discussion of the genome size of *M. thalassinum* (I found one estimate of a haploid genome of 12.72 pg, or about 12.4 Gbp, by Hawlitschek *et al*., 2023 ^1^) compared to the final assembly (~9.04 Gbp). This would naturally pose additional research questions: Is the assembly more or less complete than expected? What parts of the genome are missing from the assembly, and where are they located? These are obviously non-trivial questions to answer and are well outside the scope of a data note paper, but I would be interested to see some mention of these to provide additional context to the results.

Are sufficient details of methods and materials provided to allow replication by others?

Yes

Is the rationale for creating the dataset(s) clearly described?

Yes

Are the datasets clearly presented in a useable and accessible format?

Yes

Are the protocols appropriate and is the work technically sound?

Yes

Reviewer Expertise:

NA

I confirm that I have read this submission and believe that I have an appropriate level of expertise to confirm that it is of an acceptable scientific standard.

## References

[1] : New estimates of genome size in Orthoptera and their evolutionary implications. *PLOS ONE* .2023;18(3) : 10.1371/journal.pone.0275551 10.1371/journal.pone.0275551 PMC1001664836920952

# Reviewer report: Reviewer response for version 1

**DOI:** 10.21956/wellcomeopenres.23770.r92599

**Published:** 2024-08-29

**Authors:** Sheng-Quan Xu

**Affiliations:** 1 Shaanxi Normal University, Hanzhong, China

Most Orthoptera insects have gigantic genomes. *Meconema thalassinum*, reported in this paper is the largest known chromosome-level insect genome. Which is of great theoretical value for the study of katydid evolution and genetics, and it also provides important data for the study of the structure and composition of mega genomes.

 The paper is basically reasonable in terms of description and methodology.

**There are the following two questions to be added:**

1. Please state which data and the amount of data used for the assembly of the mitochondrial genome.
2. Label the position of chromosome 1_2 in Fig. 5.

Are sufficient details of methods and materials provided to allow replication by others?

Partly

Is the rationale for creating the dataset(s) clearly described?

Yes

Are the datasets clearly presented in a useable and accessible format?

Yes

Are the protocols appropriate and is the work technically sound?

Yes

Reviewer Expertise:

evolutionary genomics of Orthoptera insects and leeches

I confirm that I have read this submission and believe that I have an appropriate level of expertise to confirm that it is of an acceptable scientific standard.
